# Use of Nanocellulose to Produce Water-Based Conductive Inks with Ag NPs for Printed Electronics

**DOI:** 10.3390/ijms23062946

**Published:** 2022-03-09

**Authors:** Sandra Martinez-Crespiera, Belén Pepió-Tàrrega, Rosa M. González-Gil, Francisco Cecilia-Morillo, Javier Palmer, Ana M. Escobar, Sirio Beneitez-Álvarez, Tiffany Abitbol, Andreas Fall, Christian Aulin, Yuval Nevo, Valerio Beni, Enrico Tolin, Achim Bahr

**Affiliations:** 1 Applied Chemistry and Materials, ARTS Department, Leitat Technological Center, C/Pallars, 186-179, 08005 Barcelona, Spain; bpepio@leitat.org (B.P.-T.); rmgonzalez@leitat.org (R.M.G.-G.); 2 Applied Chemistry and Materials, ARTS Department, Leitat Technological Center, C/de la Innovació, 2, 08225 Barcelona, Spain; fcecilia@leitat.org (F.C.-M.); jpalmer@leitat.org (J.P.); amescobar@leitat.org (A.M.E.); 3 Advanced Engineering, ARTS Department, Leitat Technological Center, C/de la Innovació, 2, 08225 Barcelona, Spain; sbeneitez@leitat.org; 4 Bioeconomy and Health Division, RISE Research Institutes of Sweden, Drottning Kristinas väg 61, 114 28 Stockholm, Sweden; tiffany.abitbol@ri.se (T.A.); andreas.fall@ri.se (A.F.); christian.aulin@ri.se (C.A.); 5 Melodea Ltd., Rehovot 76100, Israel; yuval@melodea.eu; 6 Digital Systems, Smart Hardware, Bio- and Organic Electronics Division, RISE Research Institutes of Sweden AB, 601 17 Norrköping, Sweden; valerio.beni@ri.se; 7 IMST GmbH, 47475 Kamp-Lintfort, Germany; enrico.tolin@imst.de (E.T.); achim.bahr@imst.de (A.B.)

**Keywords:** nanocellulose, silver nanoparticles, conductive inks, sustainable printed electronics, screen-printing

## Abstract

The need for more sustainable printed electronics has emerged in the past years. Due to this, the use of nanocellulose (NC) extracted from cellulose has recently been demonstrated to provide interesting materials such as functional inks and transparent flexible films due to its properties. Its high specific surface area together with the high content of reactive hydroxyl groups provide a highly tailorable surface chemistry with applications in ink formulations as a stabilizing, capping, binding and templating agent. Moreover, NC mechanical, physical and thermal properties (high strength, low porosity and high thermal stability, respectively) provide an excellent alternative for the currently used plastic films. In this work, we present a process for the production of water-based conductive inks that uses NC both as a template for silver nanoparticles (Ag NPs) formation and as an ink additive for ink formulation. The new inks present an electrical conductivity up to 2 × 10^6^ S/m, which is in the range of current commercially available conductive inks. Finally, the new Ag NP/NC-based conductive inks have been tested to fabricate NFC antennas by screen-printing onto NC-coated paper, demonstrating to be operative.

## 1. Introduction

Over the past decade, printed electronics have become a promising field with highlighted developments over various applications [1]. This emerging field combines the development of functional inks and their deposition by conventional printing processes to produce more cost-effective and eco-friendly electronic products. A wide range of low-cost printing processes can be applied, from conventional processes such as inkjet and screen-printing, to coating procedures such as slot-die, spin-coating, or sputtering techniques [2]. These processes ensure a good level of productivity, and together with the use of sustainable substrates and inks, represent a better alternative to currently used electronic devices. 

Recently, nanocellulose (NC) has emerged in the field of printed electronics as a sustainable and green material, capable of substituting the common polymers and materials obtained from petroleum sources, thanks to its promising characteristics. Individual NC elements have extremely high stiffness (up to 14 GPa), high strength (up to 200 MPa), high specific surface area (several hundreds of m^2^/g), low density (up to 1.6 g/cm^3^), tuneable surface chemistry and good biocompatibility and biodegradability. NC free-standing films have low porosity (up to 2%), high transparency across the visible spectrum (up to 90% transmittance at 550 nm), high thermal stability (up to 300 °C) and a very low oxygen barrier (oxygen transmission rate (OTR) < 10 cm^3^/m^2^ day) [3]. NC is the nanosized structure of cellulose, which is the most abundant polymer in nature. It can be extracted from plants (wood, rice husk, sisal, hemp, flax, kenaf and coconut husk, algae, etc.), animals (tunicates) and bacteria. Three types of NC can be distinguished: cellulose nanofibers (CNF) and cellulose nanocrystals (CNC) extracted from plants and animals and bacterial nanocellulose (BNC) produced by bacteria. Cellulose nanofibers (CNF) are nanofibers with a high aspect ratio (>50) with a wide range of lengths (0.3–10 µm) and a small diameter (2–20 nm), depending on the source and the preparation method. The nanofibers are mainly isolated by mechanical processing of cellulose fibers, mainly from wood [4], providing a material with both amorphous and crystalline regions. Cellulose nanocrystals (CNC) are rod-shaped nanocrystals with a small diameter (2–20 nm) and length (100–500 nm) that are obtained by a chemical treatment (acid hydrolysis) of cellulose that eliminates the amorphous regions, forming colloidal suspensions that can be organized as a liquid crystal (in chiral nematic phase) above a certain concentration [5]. Bacterial nanocellulose (BNC) has a fibrillar structure equivalent to CNF and is produced by bacteria.

**Figure 1:**
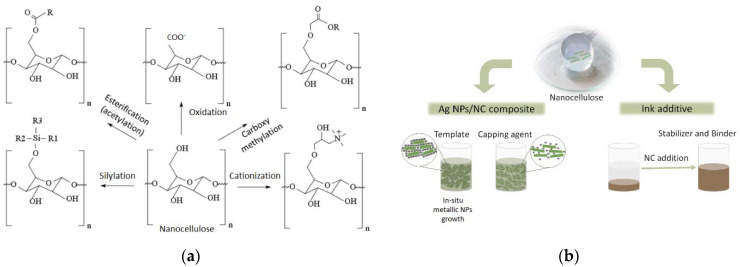
(**a**) Scheme of common modification reactions of NC. (**b**) Scheme of NC properties suitable for conductive composites and inks.

NC is hydrophilic and tends to form aggregates in non-polar solvents due to the high hydrogen bonding interactions hindering its dispersibility. However, surface hydroxyl groups can be readily modified to promote dispersion in non-aqueous media or other functional properties. Figure 1a shows the most common functionalization reactions used to modify the NC surface. In this sense, positive or negative charges can be introduced on the surface by carboxymethylation, oxidation with (2,2,6,6-Tetramethylpiperidin-1-yl)oxyl or (2,2,6,6-tetramethylpiperidin-1-yl)oxidanyl (TEMPO) or cationization, while esterification (acetylation), silylation, etc., can introduce hydrophobic moieties [6]. The introduction of these different groups makes the possible applications of NC highly versatile, for example in the development of different functional materials, such as composites and inks. NC can be used as a template and capping agent for the synthesis of nanoparticles, such as silver nanoparticles (Ag NPs), as well as a dispersing, stabilizing and binding agent for the ink formulations (Figure 1b) [7,8].

Most common metallic conductive inks contain micro- or nano-sized Cu or Ag metallic particles in a solvent (generally water and alcohols) that includes additives (generally dispersants, thixotropic and coalescing agents) to provide stability and good printability. Although Cu is a more sustainable alternative, Ag is more resistant to oxidation. There are several examples in the literature of conductive composites and inks containing NC and Ag, and we have summarized the most relevant ones in Table 1 [9,10,11,12,13,14,15,16,17,18,19]. In [10], stable silver nanoparticles (Ag NPs) were synthesized using dialdehyde cellulose nanocrystal (DACNC) as both a reducing and stabilizing agent to be used as an antibacterial additive in paper-based food packaging. Ag NPs/CNC composites were also prepared in [11] by heating AgNO_3_ in the presence of CNC to be used as a nanocatalyst for degradation of organic dyes. References [12,13,14] use TEMPO-CNC to synthesize the composites, allowing to produce conductive suspensions at a very low silver content compared to classical silver ink. It is explained how charge density is found to have a strong impact on the shape, organization and suspension stability of the resulting silver particles. To the best of our knowledge, the highest electrical conductivity reported is 2.9 × 10^6^ S/cm, from Meulendijks et al. [15], for an ink prepared in a multistep process, that achieved 94 wt.% of Ag NPs in a stable suspension. The high Ag content was attributed to the two-step process, in which the formation of new Ag NPs on the CNC surface and the growth of previously existing Ag NPs took place during the second step. In [16], a composite is also prepared for biomedical applications, with enhanced mechanical properties, electrical conductivity and antibacterial activity, by exploiting mussel’s cathecol chemistry. Thermal reduction is used in [17] to obtain the composite from BNC. In [18,19], sulfonated-CNC is used as a template to synthesize Ag NPs using NaBH_4_ and pyrrole, respectively. In [18], a correlation between the CNC surface chemistry with the size distribution of the Ag NP is demonstrated, and in [19], polypyrrole nanoparticles are also introduced by in situ polymerization of pyrrole to prepare an electroconductive composite. 

In our work, we describe the preparation of different Ag NP/NC composites produced with a cost-effective and up-scalable process, with an Ag content up to 98 wt.%. With these composites, we were able to formulate new conductive water-based inks suitable for screen-printing and with a conductivity up to 2 × 10^6^ S/m. Moreover, we have tested these new inks to fabricate NFC antennas by screen-printing onto NC-coated paper, with promising results.

## 2. Results and Discussion

### 2.1. Synthesis and Physicochemical Characterization of Ag NP/NC Composites

**Figure 2:**
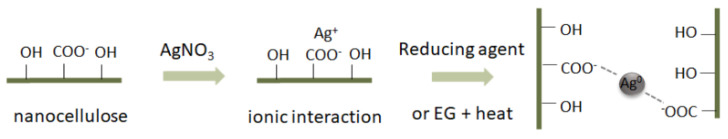
Scheme to show the interactions between Ag NPs and NC during the synthetic procedure.

Metal nanoparticles are usually prepared with bottom-up methods, through the reaction of a solution of metal salts and a reducing agent or by thermal decomposition of precursor molecules [20]. In the case of Ag NPs, they are usually synthesized by reduction in an aqueous medium, and the most used reducing agents are sodium borohydride (NaBH_4_), hydrazine, ascorbic acid and sodium citrate. Other methods include reduction in organic solvents [10], electrolysis of metal salts [21] or thermal decomposition processes [22]. In this work, Ag NPs are directly synthesized on the NC surface by reduction of Ag^+^ ions from AgNO_3_ using two different methodologies: (a) using an external reducing agent (NaBH_4_ or hydrazine), and (b) using the hydroxyl groups of the NC itself acting as a reducing agent and with ethylene glycol (EG) as the solvent and co-reducing agent at a high temperature (140 °C) [5]. In order to increase the chemical interaction (ionic) of the NC with the Ag^+^ ions and the Ag NPs afterwards, negatively charged carboxylic groups were introduced onto the NC surface. For this, two different methodologies were used: the carboxymethylation reaction for CNF and the TEMPO-mediated oxidation for CNC. In both cases, the extent of functionalization was quite high, with an oxidation degree (DO) of about 0.3 (corresponding to 3 carboxylic groups per 10 glucose units, measured by conductometric titration against NaOH). This high DO generates a high degree of charges onto the NC surface that enhances its colloid stability and dispersibility in aqueous media. The presence of these carboxylic groups on the NC surface promotes interactions with Ag ions and enables a homogeneous distribution across its surface (Figure 2).

**Figure 3:**
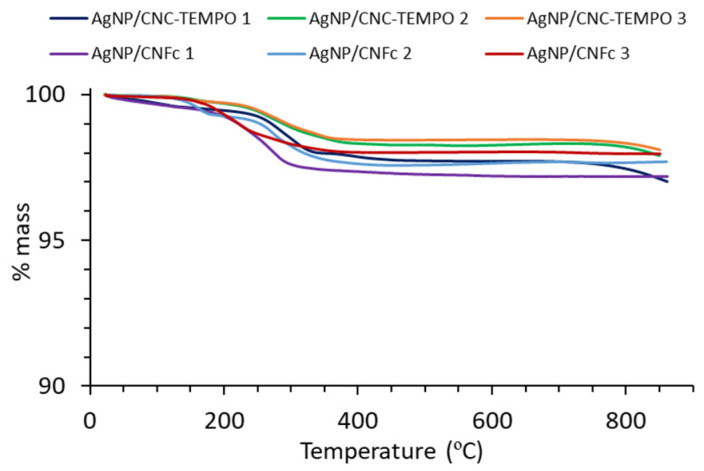
Thermogravimetric (TGA) measurements of Ag NP/NC composites.

Table 2 summarizes all the synthesized Ag NP/NC composites, including their method of synthesis and the Ag content (wt.%, *p*/*p*). A high Ag NPs content, up to approximately 98 wt.%, was reached in all cases, with no obvious difference either in the reducing method or the NC type. The Ag content of each Ag NP/NC composite was evaluated by thermogravimetric analysis (TGA, Figure 3). From the TGA, it was observed for all samples that the most relevant mass loss started at approximately 200 °C until approximately 350 °C, which corresponds to the nanocellulose degradation. In case of samples Ag NP/CNC-TEMPO 1 and Ag NP/CNFc 1, a small mass loss (less than 0.5 wt.%) was also observed at approximately 100 °C, which would correspond to residual water. From these results, it is concluded that in all cases, a high Ag content was achieved (97–98 wt.%), being the highest ever reported in the literature, to the best of our knowledge, for Ag NP/NC composites for conductive inks.

**Figure 4:**
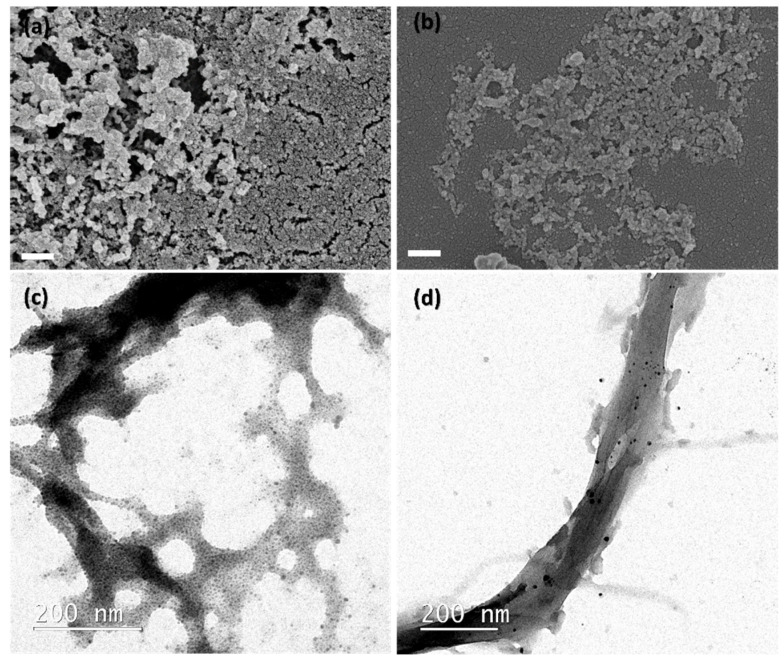
SEM and TEM images from (**a**) Ag NP/CNC-TEMPO 3, (**b**) Ag NP/CNFc 2, (**c**) Ag NP/CNC-TEMPO 1 and (**d**) Ag NP/CNFc 3. Scale bars from SEM images are 300 nm.

The high capacity of the NC to complex nearly all added Ag and well-distributed Ag NPs covering the entire surface of the NC can be observed in the SEM and TEM images (Figure 4). In case of the SEM images, a high concentration of the Ag NPs can be observed agglomerated around the nanofibrils (in case of CNF) or nanorods (in case of CNC). For the TEM images, this is better shown, since nanofibrils and nanorods can be better observed. No significant difference in nanoparticle size was observed when varying the reducing agent (5–10 nm) (Table 2).

**Figure 5:**
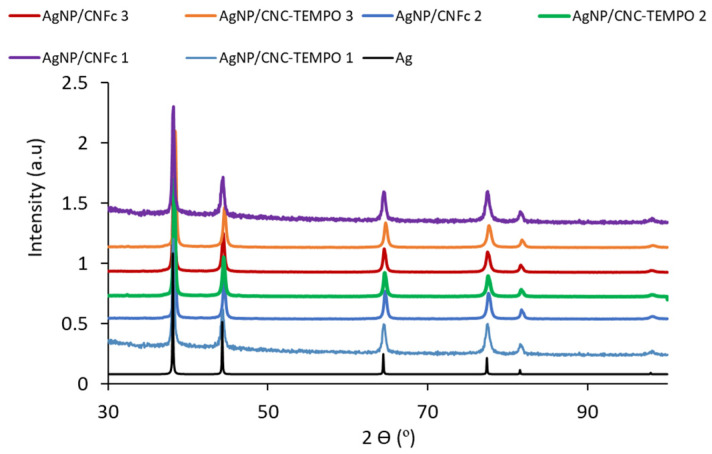
XRD patterns of as-synthetized Ag NP/NC composites. FCC crystalline Ag pattern has been added for comparison purposes.

Finally, X-ray diffraction (XRD) and X-ray photoelectron spectroscopy (XPS) analysis were performed to ensure the metallic nature of the as-synthetized Ag NPs. XRD analysis (Figure 5) showed peaks for all composites at 2θ = 38°, 44°, 64°, 78° and 82° corresponding to the planes (111), (200), (220), (311) and (222) of the face-centered cubic (FCC) crystalline metallic Ag NPs phase, respectively. The presence of silver oxides was not observed. The high concentration of Ag NPs in the composites obscures the signals corresponding to the NC. These results agree with the ones obtained by XPS for Ag NP/CNFc1. It is known that small binding energy shifts were observed for Ag^+^ compounds, more pronounced in the Auger lines (~2.5 eV shift). The Auger parameter is a powerful method to identify chemical states of the elements. Essentially, this parameter is the sum of the binding energy of the most intense photoelectron peak and the kinetic energy of the sharpest Auger peak. Supplementary Materials Figure S1 shows kinetic energy for Ag_MNN_ and binding energy for Ag3d. Taking these into account, the calculated Auger parameter of the analyzed sample was 726.2, which corresponds to metallic Ag [23].

### 2.2. Ink Formulation and Characterization

Conductive screen-printing inks are generally composed of three components: the conductive (nano)particles, the solvent and the additives (including a binder). In our case, and as commented on in the Experimental Section, our inks contain the Ag NP/NC composite in a water/isopropyl alcohol (IPA) solvent with several additives (dispersing agent DISPERBYK 2012, thickener, rheological agents Reobyk 7420 and hydroxypropyl methyl cellulose (HPMC) and HCl). A suitable screen-printing ink needs to have a high viscosity (1000–10,000 cP) and therefore, a high solid content is generally required (40–60 wt.%). In this work, we aimed for an approximately 50 wt.% Ag NPs content in the final formulation, to have a high conductivity without compromising its printability, and the amount of solvent and additives were adjusted for an appropriate dispersion and printability. Table 3 summarizes the different conductive inks formulated with the Ag NP/NC composites. Values of electrical conductivity up to 2 × 10^6^ S/m were reached on the ITEMPO3 ink, which are, to the best of our knowledge, the highest values reported for NC-based conductive inks using a one-step process for the Ag NP/NC composite. It is observed that, generally, the inks containing CNC-TEMPO are more conductive than the ones with CNFc. This has already been observed in the literature by several authors (Table 1). A possible explanation could be that Ag NPs are better interconnected when CNC is used due to the size differences. The long flexible CNFs wrap the Ag NPs; meanwhile, for CNC, the Ag NPs protrude out from the CNC nanorod surface. This better interconnection favors a higher electrical conductivity. Higher values are obtained using NaBH_4_ or thermal treatment with ethylene glycol (EG) as reducing agents. In case of ICNFc1, the conductivity is lower due to the lower content on Ag NPs in the formulated ink (48.6 wt.% vs. 53–54 wt.% for the rest).

**Figure 6:**
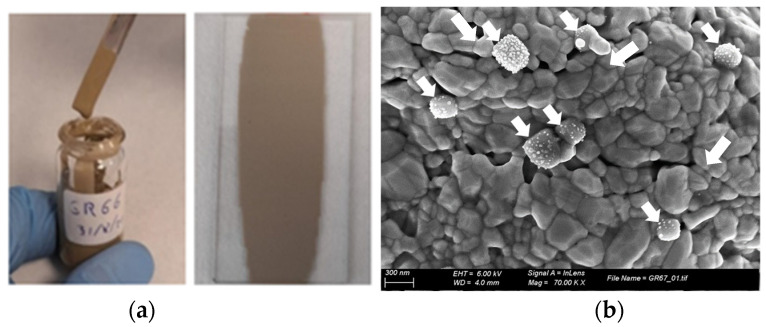
(**a**) Images of the formulated and printed ink by bar coating onto a glass substrate, and (**b**) SEM image of the ITEMPO3 ink after sintering at 150 °C for 30 min scaled at 300 nm, arrows mark the smaller Ag NPs (right).

The physical aspect of the formulated inks and the deposition onto a glass substrate by bar coating (50 µm wet) is shown in Figure 6a. Well-dispersed homogeneous inks were obtained and their deposition onto glass provided films with good adhesion and homogeneity. The SEM images of the ink ITEMPO3 after the sintering at 150 °C for 30 min show the agglomerated Ag NPs all connected between each other, which is important for the electrical conductivity. Moreover, some smaller Ag NPs can be observed, which correspond to the observed Ag NPs in the TEM images shown in Figure 6b, similar to results obtained in [12].

**Figure 7:**
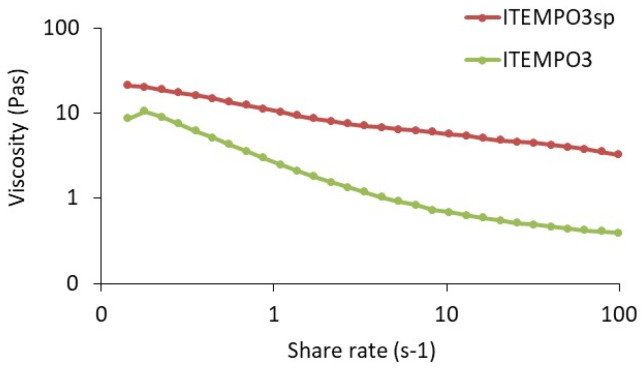
Rheological study (shear rate vs. viscosity) of the new formulated conductive inks.

Rheological studies were performed on the ITEMPO3 ink, which as mentioned exhibits the highest conductivity (Figure 7). This ink needed an optimization for its printability by screen, and therefore the amount of additive (hydroxypropyl methyl cellulose) was increased in order to adjust the viscosity to the screen-printing requirements. We present the rheological curve for the base ITEMPO3 ink and for the screen-printing-optimized ink (ITEMPO3sp), presenting a higher viscosity value at elevated shear rates.

### 2.3. Screen-Printing and NFC Performance Evaluation

**Figure 8:**
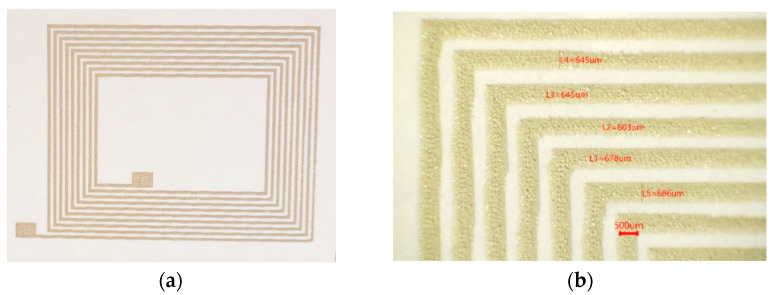
(**a**) Printed NFC antenna onto CNC-coated Klabin substrate and (**b**) magnification of the printed lines with the optical microscope.

As mentioned in Section 2.2, the ITEMPO3sp ink was formulated to improve its printability onto paper (CNC-coated Klabin substrate), but therefore producing a slight decrease in the electrical conductivity. NFC antennas were printed onto NC-coated paper using the new formulated inks with good results (Figure 8a). Printed lines’ resolution was evaluated using an optical microscope, showing a resolution down to 600 µm (Figure 8b). The thickness measurement by profilometry of the printed lines of the NFC antenna tracks was between 15 and 25 microns with a single screen-printer impression pass. 

For the NFC antenna performance evaluation, six printed samples have been measured. In the preliminary series, three samples were discarded due to discontinuity and short-circuits in antenna tracks. The impedance measurements of the three remaining samples (ITEMPO3sp-1, 2 and 3) can be seen in [12]. The values have been extracted and calculated from the input impedance of the antenna starting at 1 to 70 MHz (Table 4).

The results show that the Q-factor does not match the minimum required value (>10) of industry specification ISO14443 mainly due to the poor conductivity of the ink. A low Q-factor value means a short reading distance of the NFC antenna. However, the value of the inductance is good enough for the antenna to be operative. In order to improve the Q-factor and diminish the reading distance (now 2.5 cm), a higher ink conductivity is needed (10^7^ S/m). Another aspect to take into account for improvement is the repeatability and resolution of the printing process, since only 50% of the samples have had no failure.

## 3. Materials and Methods

All reagents were used as received. Commercial sulfite softwood pulp was from Domsjö Dissolving plus; Domsjö Fabriker AB, Domsjö, Sweden. Ethanol (Rectapur^®^) was purchased from VWR (Stockholm, Sweden). CNC nanocellulose was provided by Melodea Bio-Based Solutions (Rehovot, Israel). Monochloroacetic acid (99%, ACS reagent), acetic acid (ACS reagent), Iso-propanol (ACS reagent), sodium hydroxide (ACS reagent), sodium hydrogen carbonate (ACS reagent) and methanol (ACS reagent) were purchased from Sigma Aldrich (Stockholm, Sweden). CNC was provided by Melodea Ltd. 2,2,6,6-Tetramethylpiperidine 1-oxyl (TEMPO, 98%, free radical), sodium bromide (ACS reagent, ≥99.0%), sodium hypochlorite solution ((6–14% active chlorine) EMPLURA^®^, Supelco) and hydrazine hydrate (Reagent grade, 50–60%) were received from Sigma Aldrich (Madrid, Spain). Silver nitrate (ACS 99.9+%, metals basis) and sodium borohydride (<98%) were purchased from Alfa Aesar (Barcelona, Spain). Ethylenglycol (EG, ExpertQ^®^, Reag. Ph Eur) and ethanol (96% *v*/*v*, Pharmpur^®^, Ph Eur, BP) were purchased from Scharlau (Barcelona, Spain).

### 3.1. Synthetic Procedures

#### 3.1.1. Procedure to Obtain Carboxymethylated CNF

The carboxymethylated CNF was produced first by carboxymethylation of the dissolving pulp followed by high-pressure homogenization according to Wågberg et al. [24]. The DO was increased to 0.3 following Rosén et al. [25] by increasing the amounts of reacting agent (monochloroacetic acid) and sodium hydroxide. Here, 12 g of both per 10 g of dry weight of pulp was used to reach a DO of 0.3. Finally, the modified fibers were homogenized once using a high-pressure fluidizer (Microfluidizer M-110EH, Microfluidics Corp., Westwood, MA, USA) operating at 1700 bar to fibrillate the pulp and produce the CNFc.

#### 3.1.2. Procedure to Obtain CNC-TEMPO

Cellulose nanocrystals were produced by Melodea Bio-Based Solutions (Rehovot, Israel) and subjected to TEMPO-mediated oxidation to introduce carboxylate groups. TEMPO-mediated oxidation of CNC was performed using a previously described procedure in the literature [26]. Then, 166 g of CNC at 3 wt.% was dispersed in 150 mL of distilled water and subjected to ultrasonic probe sonication for 10 min (Pulses of 1″ and 40% amplitude). Then, an aqueous solution (100 mL) of TEMPO (85 mg) and NaBr (530 mg) was added to a suspension of CNC, and a solution of NaOCl (50–60% active, 35.3 mL, 10 eq. with respect to CNC) was added slowly while maintaining the pH at 10 using a 1M NaOH solution. After 16 h, the reaction was ended by adding ethanol (100 mL), and the pH was adjusted to 7 by washing/centrifugation with a mixture of ethanol and water (7:3) (20,000 rpm, 10 min). CNC-TEMPO (5.3 wt.%) was stored in the fridge before use. 

#### 3.1.3. Procedure to Obtain Ag NP/NC Composite

In a typical procedure, 50 g of CNFc dispersion in water (1 wt.%), or 9.4 g in case of CNC-TEMPO dispersion (5.4 wt.%), were mixed with a total amount of water of 150 mL and well-homogenized using an Ultraturrax homogenizer (IKA, S-18N 19G) at 15,000 rpm, and then a tip sonication (ultrasonic processor HIELSCHER, UIP1000HD model) at 100% amplitude. Afterwards, an aqueous solution of AgNO_3_ (40 g, 130 mL) was added to the previous NC dispersion and homogenized at 10,000 rpm with Ultraturrax for 5 min and sequentially sonicated by a tip ultrasonication for 1 h, using an ice-water bath to avoid overheating. In the case of using an external reducing agent, the reducing agent was dissolved in water (8.9 g of NaBH_4_ in 130 mL of H_2_O, or 14 mL of hydrazine hydrate in 50 mL of H_2_O), and then it was added dropwise to the Ag NC solutions under ultrasonication. After the addition, the mixture was sonicated for one more hour at room temperature. In a final step, the mixture was centrifuged at 10,000 rpm for 10 min to separate the Ag NP/NC composite from the soluble byproducts and unspent reagents. The obtained pellet was redispersed in 1000 mL of distillated water and stirred for 16 h at room temperature, followed by tip sonication to obtain a homogeneous dispersion. The drying step was carried out using a spray-dryer (BÜCHI, Mini spray-dryer B-290) with an inlet temperature of 197 °C, a gas flow of 50% and a pump rate of 9 mL/min. Finally, the collected powder was ball-milled (Fritsch Premium Line, Pulverisette) to homogenize the final Ag NP/NC composite (5 cycles at 600 rpm during 10 min each cycle). In the case of the thermal reduction, the procedure was very similar but using EG as a solvent. First, the NC dispersions were mixed with 200 mL of EG and homogenized using an Ultraturrax at 15,000 rpm, and then a tip ultrasonication at 100% amplitude. Afterwards, the AgNO_3_ was dissolved in EG (150 mL) and added to the previous NC solution, followed by a homogenization step by Ultraturrax and ultrasonication at 100% amplitude for 2 h. The mixture was left overnight at 140 °C under magnetic stirring. Then, the mixture of Ag NP/NC was treated in the same way as the non-thermal process, followed by centrifugation, spray-drying and ball-milling, respectively.

#### 3.1.4. Procedure for Ink Formulation

The screen-printing formulations of the Ag conductive inks (10 g/batch) were produced from a mixture of 40–60 wt.% of the Ag NP/NC composite (with 96–98 wt.% Ag NPs) in 15–25 wt.% of distilled water and 8–18 wt.% of isopropyl alcohol (IPA). Moreover, a dispersing agent (Disperbyk 2012 1–5 wt.%) was added to avoid particle aggregations and flocculation. The resulting suspension was homogenized in an ultrasonic sonicator (Sonics model VCX 750) for 1 h in an ice bath for a correct dispersion of the nanoparticles. Afterwards, the mixture was agitated in a Dispermat (VMA-GETZMANN GMBH model CV3) for another hour at 1000 rpm. Then, several components were added: 7–20 wt.% of hydroxypropyl methylcellulose (HPMC) as a thickening and suspending agent, a rheological additive (Reobyk 7420) to enhance the thixotropic effect (1–5 wt.%) and 0.5–2 wt.% of HCl 1N to enhance the Ag NPs coalescence during the sintering step.

#### 3.1.5. Screen-Printing Procedure

The formulated inks were printed in the form of an NFC antenna using a screen-printer Aurel C920. The antenna design was integrated in a water-compatible 61/64/100 screen mesh. NC-coated paper (CNC-coated Klabin) was used as a substrate, previously treated in a hot press at 120 °C for 15 min to improve film properties. The screen-printing conditions were optimized for proper transferring of the ink through the screen mesh, using a gap distance from the screen to the substrate of 1–2 mm, with a squeegee speed and pressure of 15–80 mm/s and 2–8 kg/cm^2^, respectively. Finally, the printed antennas were cured in air atmosphere for 30 min at 150 °C.

### 3.2. Characterization Methods

#### 3.2.1. Conductimetric Titration

The oxidation degree (DO) obtained after TEMPO-mediated oxidation was performed using an automatic conductimetric titrator METROHM (TITRANDO 888 model). Then, 40 mg of dry CNC-TEMPO sample was dispersed in 1 mM NaCl solution by tip ultrasonication (10 min, 1″ pulses, 40% amplitude), adjusted with HCl 1M to pH = 2–3, and titrated against NaOH 0.1 M at a rate of 0.1 mL min^−1^ using 15 s intervals. The DO for the carboxymethylated sample was also determined by conductometric titration. The measurement was performed on the modified pulp before the fibrillation by high-pressure homogenization, following Katz et al. [27]. For both the TEMPO and carboxymethylated NC, the DO was determined by the equation below:

$$\mathrm{DO}=\frac{162\cdot \left(C_{\mathrm{NaOH}}\cdot \left(V_{2}-V_{1}\right)\right)}{m_{\mathrm{NC}}-x\cdot {\;C}_{\mathrm{NaOH}}\cdot \left(V_{2}-V_{1}\right)}$$

x(TEMPO) = 38   x(carboxymethylation) = 80

where C_NaOH_ is the concentration of NaOH (M), V_i=1,2_ stands for the volume of NaOH (L) in the conductimetric titration curve, x is the difference in the molecular weight for the oxidated NC and the non-oxidated NC and m_NC_ is the weight of dry NC (g).

#### 3.2.2. Thermogravimetric Analysis (TGA)

The amount of Ag in the Ag NP/NC composites was quantified by TGA (TA Instruments Q500) using a heating rate of 20 °C/min from room temperature to 900 °C under air conditions, and the final residue was assigned to the total metal content with an estimated error value of approximately 0.05%.

#### 3.2.3. Scanning Electron Microscopy (SEM)

SEM images were obtained using a ZEISS MERLIN high-resolution field emission scanning electron microscope (FE-SEM) using both secondary electrons (SE) and backscattered electrons (BSE) (Electron Microscopy Facilities from Universitat Autonòma de Barcelona (UAB, Bellaterra, Spain)). Samples were previously metalized with 4 nm of gold (K500X Manual Sputter Coater de Quorum Technologies). The beam voltage was set to 4 kV and the working distance to 4.4 mm. Samples were prepared by dispersing the Ag NP/NC composite powders in distilled water by tip ultrasonication until a homogeneous dispersion was achieved. Then, samples were deposited by drop-casting onto carbon substrates. SEM images were taken to examine the composites’ morphology, whereas BSE images were taken to identify the Ag NPs on the surface.

#### 3.2.4. Transmission Electron Microscopy (TEM)

TEM images were obtained using a Jeol JEM-1400 Transmission Electron Microscope from Electron Microscopy Facilities from Universitat Autonòma de Barcelona (UAB, Bellaterra, Spain). Samples were previously redispersed in distilled water by ultrasonication, and then drop-casted onto a copper carbon grid. Ag NPs’ size was analyzed with Imagej software. The average diameter was manually calculated adjusting the scale. For all samples, N > 100. 

#### 3.2.5. X-ray Diffraction (XRD)

XRD profiles were obtained with the PANalytical X’pert PRO MRD (Multipurpose Diffractometer) (Malvern PANalytical, Düssseldorf, Germany) equipped with a CuKα radiation source (λ = 1.5406 Å), from the X-ray Diffraction Facility from Institut Català de Nanociència (ICN2, Bellaterra, Spain). This diffractometer has a vertical theta-theta goniometer (240 mm radius). The XRD analysis was carried in reflexion mode with a lineal X’Celerator detector for the diffracted beam, from 10 to 100° (2 theta), with a step size of 0.05 and a counting time of 250 s. XRD was performed on the modified CNC as well as the Ag NP/NC composites. Patterns were obtained to check the crystallinity.

#### 3.2.6. X-ray Photoelectron Spectroscopy (XPS)

XPS measurements were conducted at the Photoemission spectroscopy Facility (Institut Català de Nanociència, ICN2, Bellaterra, Spain) at room temperature with a SPECS PHOIBOS 150 hemispherical analyzer (SPECS GmbH, Berlin, Germany) at a base pressure of 5 × 10^−10^ mbar, using monochromatic Al K alpha radiation (1486.74 eV) as the excitation source operated at 300 W. The energy resolution as measured by the FWHM of the Ag 3d5/2 peak for a sputtered silver foil was 0.62 eV. The spectrum was calibrated with respect to the C1s at 284.8 eV.

#### 3.2.7. Conductivity and Resistivity Measurements

The conductivity of the composites was measured by a Cylindrical Four-Point probe (Jandel Engineering) [5], a technique in which four conductive tips are in contact with the sample, aligned and at a known distance from one another. A known current was applied between the two outer tips, and the voltage difference between the inner tips was measured, from which the sheet resistivity of the material can be calculated:

$$\rho_{s}=\frac{\pi V}{\ln\left(2\right)I}$$

where $\rho_{s}$ is the sheet resistivity (Ω/square), V is the voltage (V) and I is the current (A). The sheet resistivity can be converted to bulk resistivity by multiplying by the thickness of the sample.

#### 3.2.8. Thickness Layer

The thickness of the layers and the line widths obtained by bar-casting and screen-printing were evaluated using a mechanical profilometer (ALPHA STEP D 600 3D, KLA-TENCOR) at 0.2 mm/s.

#### 3.2.9. Viscosity

Ink viscosity was measured using a BOHLIN CVO 120 rheometer. A sample of 1–2 mL was subjected to an increasing shear rate from 0.1 to 100 s^−1^. The measure of the shear stress with respect to the shear rate provides the viscosity curve (Pa·s).

#### 3.2.10. Procedure for NFC Antenna Evaluation

The antenna design used in this work is a loop antenna, and it should resonate at 13.56 MHz according to industrial standard ISO/IEC 18000-3:2010. The printed NFC antennas were measured by a network analyzer, N9914A FieldFox Handheld RF Analyzer, in order to validate the requirements. The serial resistance and inductance of the NFC coils were measured at the NFC frequency of 13.56 MHz. The NFC antenna samples were connected to the impedance analyzer using spring-loaded connectors directly on the traces. To minimize the effects of the connections, open-circuit and short-circuit calibration were performed for all connections before measuring. Series-resistant measurement was performed by a Fluke 115 True Rms multimeter. The antenna model used for the characterization follows these values: shape = rectangular, total size = 40 × 56 mm, track width = 0.7 mm, gap width = 0.4 mm, goal inductance = 5 uH.

## 4. Conclusions

In this work, we have demonstrated the potential use of NC to produce water-based conductive inks with a high electrical conductivity (up to 2 × 10^6^ S/m), useful for the fabrication of printed electronics. The NC acts both as a template for the Ag NPs’ synthesis and as a stabilizing agent for the final conductive inks. The two types of NC used, CNFc and CNC-TEMPO, are suitable for the Ag NP/NC composite synthesis, with higher conductivity results achieved when using the CNC-TEMPO. The better interconnectivity between Ag NPs in CNC nanorods vs. CNF nanofibrils could explain these results. The use of different reduction processes affords similar results regarding Ag NPs content onto NC, where the quantitative adsorption of nearly all the Ag by the NC was observed (up to 98 wt.%). Moreover, the formed Ag NPs (5–10 nm) were homogeneously dispersed on the NC surface, as shown by electron microscopy images. These new conductive inks can be used for screen-printing processes and are produced using water and environmentally friendly solvents (EG, IPA), which is interesting for the development of more sustainable printed electronics. As a proof of concept, NFC-printed antennas have been fabricated onto paper-based substrates (NC-coated Klabin) by a screen-printing process, providing an operative NFC antenna. This article demonstrates that functional nanocellulose can open new opportunities to develop the future generation of green materials for printed electronics.

## Figures and Tables

**Table 1:** Summary of the most relevant Ag NP/NC composites and inks found in the literature.

Type of NC	Reducing Method for Ag NP Synthesis	Ag NPs (wt.%)	Conductivity (S/m) (Sintering Conditions)	Ref.
Dialdehyde-CNC	Thermal reduction with acid/base	up to 50	*	[10]
CNC	Thermal reduction (65 °C)	39.4	*	[11]
TEMPO-CNC	Reduction with NaBH_4_	70.7	2.5 × 10^4^ (180 °C, 30 min)	[12,13]
TEMPO-CNC	Reduction with NaBH_4_	up to 97	*	[14]
TEMPO-CNC	Reduction with SnCl_2_, glucose and tartaric acid	94	2.9 × 10^6^ (450 °C, photonic)	[15]
TEMPO-CNF	Reduction with dopamine	6.8	4 × 10^4^	[16]
TEMPO-BNC	Thermal reduction (100 °C)	*	*	[17]
Sulfonated-CNC	Reduction with NaBH_4_	*	*	[18]
Sulfonated-CNC	Reduction with pyrrole	45	10^−1^	[19]

* Values not available.

**Table 2:** Summary of all synthesized Ag NP/NC composites.

Composite	Reducing Agent	Ag (wt.%)	Ag NPs Size (nm)
Ag NP/CNFc 1	NaBH_4_	97.2	9.2 ± 5.0
Ag NP/CNFc 2	Hydrazine	97.7	6.9 ± 1.8
Ag NP/CNFc 3	Thermal reduction at high T in EG	97.9	7.9 ± 2.5
Ag NP/CNC-TEMPO 1	NaBH_4_	97.0	5.0 ± 2.0
Ag NP/CNC-TEMPO 2	Hydrazine	97.9	6.5 ± 1.0
Ag NP/CNC-TEMPO 3	Thermal reduction at high T in EG	98.1	4.5 ± 2.2

**Table 3:** Prepared inks’ solid composition and their electrical conductivity.

Inks	Ag NP/NC Composite	Ag NPs (wt.%)	NC (wt.%)	Conductivity (S/m)
ICNFc1	Ag NP/CNFc 1	48.6	1.4	5 ± 0.3 × 10^4^
ITEMPO1	Ag NP/CNC-TEMPO 1	53.1	1.9	1.1 ± 0.03 × 10^6^
ICNFc2	Ag NP/CNFc 2	53.7	1.3	2.3 ± 0.5 × 10^5^
ITEMPO2	Ag NP/CNC-TEMPO 2	53.8	1.2	5.3 ± 1.3 × 10^5^
ICNFc3	Ag NP/CNFc 3	53.9	1.1	6.9 ± 0.6 × 10^5^
ITEMPO3	Ag NP/CNC-TEMPO 3	53.9	1.1	2 ± 0.06 × 10^6^

**Table 4:** Measurements of performance of printed NFC antennas.

Sample	Resistance (Ω)	Inductance (µH)	Self-Capacitance (pF)	Q Factor
ITEMPO3sp-1	113.9 ± 1.03	4.69 ± 6.94 × 10^−4^	18.20 ± 2.64 × 10^−2^	3.51 ± 0.03
ITEMPO3sp-3	64.2 ± 0.58	4.80 ± 6.94 × 10^−4^	17.79 ± 2.64 × 10^−2^	6.37 ± 0.06
ITEMPO3sp-6	46.9 ± 0.42	4.72 ± 6.94 × 10^−4^	17.98 ± 2.64 × 10^−2^	8.57 ± 0.08

## Data Availability

Not applicable.

## Supplementary Materials

The following supporting information can be downloaded at: https://www.mdpi.com/article/10.3390/ijms23062946/s1.
