# Supplementary material for: Use of Nanocellulose to Produce Water-Based Conductive Inks with Ag NPs for Printed Electronics
Source: Int J Mol Sci. 2022 Mar 9;23(6):2946. doi: 10.3390/ijms23062946 (PMC8955796; doi:10.3390/ijms23062946)
Supplement: Supplementary file 1 [file ijms-23-02946-s001.zip › ijms-1623015-supplementary.pdf]

Article

Use of Nanocellulose to Produce Water-Based Conductive Inks with Ag NPs for Printed Electronics

Sandra Martinez-Crespiera [1,*], Belén Pepió-Tàrrega [1], Rosa M. González-Gil [1], Francisco Cecilia-Morillo [2], Javier Palmer [2], Ana M. Escobar [2], Sirio Beneitez-Álvarez [3], Tiffany Abitbol [4], Andreas Fall [4], Christian Aulin [4], Yuval Nevo [5], Valerio Beni [6], Enrico Tolin [7] and Achim Bahr [7]

1 Applied Chemistry and Materials, ARTS Department, Leitat Technological Center, C/Pallars, 186-179, 08005 Barcelona, Spain; bpepio@leitat.org (B.P.-T.); rmgonzalez@leitat.org (R.M.G.-G.)
2 Applied Chemistry and Materials, ARTS Department, Leitat Technological Center, C/de la Innovació, 2, 08225 Barcelona, Spain; fcecilia@leitat.org (F.C.-M.); jpalmer@leitat.org (J.P.); amescobar@leitat.org (A.M.E.)
3 Advanced Engineering, ARTS Department, Leitat Technological Center, C/de la Innovació, 2, 08225 Barcelona, Spain; sbeneitez@leitat.org
4 Bioeconomy and Health Division, RISE Research Institutes of Sweden, Drottning Kristinas väg 61, 114 28 Stockholm, Sweden; tiffany.abitbol@ri.se (T.A.); andreas.fall@ri.se (A.F.); christian.aulin@ri.se (C.A.)
5 Melodea Ltd., Rehovot 76100, Israel; yuval@melodea.eu
6 Digital Systems, Smart Hardware, Bio- and Organic Electronics Division, RISE Research Institutes of Sweden AB, 601 17 Norrköping, Sweden; valerio.beni@ri.se
7 IMST GmbH, 47475 Kamp-Lintfort, Germany; enrico.tolin@imst.de (E.T.); achim.bahr@imst.de (A.B.)
* Correspondence: sandramartinez@leitat.org

In this supplementary information it is shown the XPS analysis of the sample CNFc1. The auger parameter is calculated by adding the binding energy of the most intense photoelectron peak with the kinetic energy of the sharpest Auger peak [Figure S1].

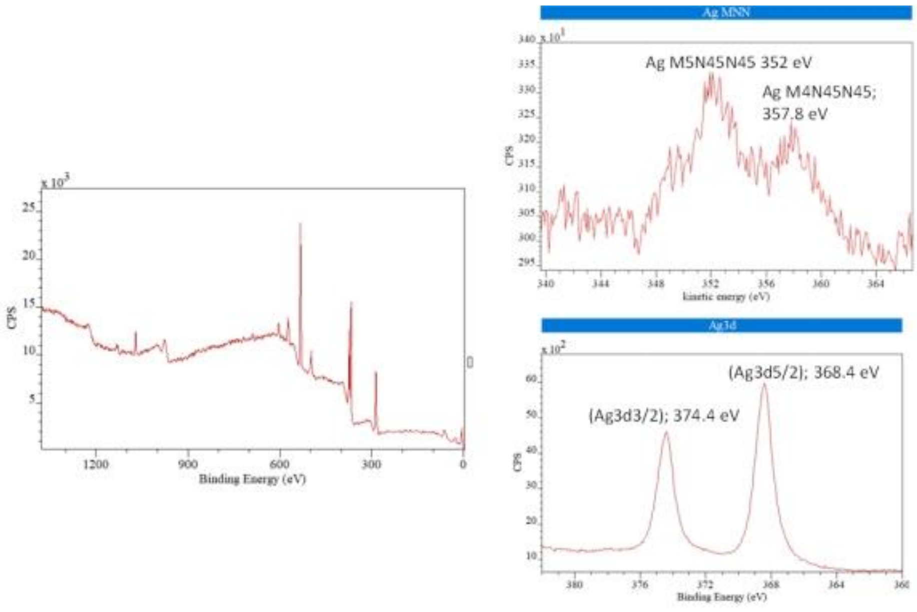

Figure S1: XPS analysis of CNFc1

Reference

S1. NIST Standard Reference Database 20, Version 4.1. Available online: https://srdata.nist.gov/xps/ (accessed on 10th February 2022).
